# Identification and Characterization of MicroRNAs from Barley (*Hordeum vulgare* L.) by High-Throughput Sequencing

**DOI:** 10.3390/ijms13032973

**Published:** 2012-03-06

**Authors:** Shuzuo Lv, Xiaojun Nie, Le Wang, Xianghong Du, Siddanagouda S. Biradar, Xiaoou Jia, Song Weining

**Affiliations:** State Key Laboratory of Crop Stress Biology in Arid Areas, College of Agronomy and Yangling Branch of China Wheat Improvement Center, Northwest A&F University, Yangling 712100, Shaanxi, China; E-Mails: lysz@nwsuaf.edu.cn (S.L.); small@nwsuaf.edu.cn (X.N.); arvin_genius@yahoo.com.cn (L.W.); xianghongdu@nwsuaf.edu.cn (X.D.); siddureddy2988@yahoo.com (S.S.B.); jiaxiaoou2005@yahoo.com.cn (X.J.)

**Keywords:** barley, miRNA, Solexa sequencing, qRT-PCR, abiotic stress

## Abstract

MicroRNAs (miRNAs) are a class of endogenous RNAs that regulates the gene expression involved in various biological and metabolic processes. Barley is one of the most important cereal crops worldwide and is a model organism for genetic and genomic studies in Triticeae species. However, the miRNA research in barley has lagged behind other model species in grass family. To obtain more information of miRNA genes in barley, we sequenced a small RNA library created from a pool of equal amounts of RNA from four different tissues using Solexa sequencing. In addition to 126 conserved miRNAs (58 families), 133 novel miRNAs belonging to 50 families were identified from this sequence data set. The miRNA* sequences of 15 novel miRNAs were also discovered, suggesting the additional evidence for existence of these miRNAs. qRT-PCR was used to examine the expression pattern of six randomly selected miRNAs. Some miRNAs involved in drought and salt stress response were also identified. Furthermore, the potential targets of these putative miRNAs were predicted using the psRNATarget tools. Our results significantly increased the number of novel miRNAs in barley, which should be useful for further investigation into the biological functions and evolution of miRNAs in barley and other species.

## 1. Introduction

MicroRNAs (miRNAs) are a class of short, highly conserved families of non-coding RNA. Most of the mature miRNAs are of 21–25 nucleotides (nt) in length and evolutionarily conserved in eukaryotes [1]. They act as important regulators either by degrading target mRNAs at post-transcriptional process or by repressing target gene translation [2]. Along with siRNA (small interfering RNA), miRNAs are also essential for cell development, differentiation, morphogenesis, signal transduction, and showed adaptive response to diverse biotic and abiotic stresses. After the discovery of the first miRNA, lin-4 in *Caenorhabditis elegans* [3], various miRNAs have been identified in diverse species of living organisms, including plants. It is estimated that miRNAs account for ~1% of predicted genes in higher eukaryotic genomes and around 10–30% of genes might be regulated by miRNAs [4].

The first plant miRNA was identified in *Arabidopsis* [5], followed by many in other species. Approximately 5000 plant miRNAs have been deposited in the miRBase18.0 database, including 291 from *Arabidopsis*, 581 from rice, 172 from maize, 44 from wheat, 171 from *Sorghum* and 142 from *Brachypodium*. Up to date in this database, only 22 miRNA from barley were identified either through experimental or computational approach. The experimental approach is to clone and sequence the small RNA library which facilitates the discovery of not only conserved but also species-specific miRNAs even if the entire genome information is not available [6]. It is not surprising that high-throughput identification of plant miRNA was made possible with the advent of next generation sequencing technologies such as 454 pyrosequencing [7] and Solexa sequencing technology [8], with a large number of plant species investigated for miRNA discovery during recent years [9–11].

Barley (*Hordeum vulgare* L.) is one of the most important cereal crops, which ranks fourth in terms of production and cultivation all over the world [12]. Furthermore, it is also well-studied in terms of genetics, genomics, and breeding and thus qualifies as a model plant for Triticeae research [13]. The exploration of miRNAs in barley has been reported through computational approach to find homologs in barley EST library [14] or by means of next-generation sequencing technology [15]. But, compared with the number of miRNAs have been identified in other cereal crops such as rice and maize, the discovery of barley miRNAs seems not to be adequate. Furthermore, identification of barley miRNA by sequencing approach has been limited to only one type of tissue (leaf) till now [15]. For mining more barley miRNAs, we constructed a small RNA cDNA library using pooled RNA from four different tissues (roots, stems, leaves and spikes) at various developmental stages (seedling, jointing, heading, filling). miRNAs were identified by sequencing this library using the Solexa deep sequencing technology combined with bioinformatics analysis. The goal of this study is to obtain a comprehensive barley miRNA profile, which will shed more light on their role in biological function and evolution.

## 2. Results and Discussion

### 2.1. Sequencing Barley Small RNA Library

We used the Solexa technology for deep sequencing of small RNA library to identify candidate miRNAs in barley. To increase the coverage of barley miRNA, we constructed a pooled small RNA library by mixing equal concentration of RNA isolated from different tissues at various developmental stages (as mentioned in Material and Methods). Solexa sequencing of this barley small RNA library was performed and a total of 10,495,264 sequence reads were obtained. After filtering out low quality tags, trimming adaptors and cleaning up shortages and contamination formed by adaptor-adaptor ligation, we obtained 9,540,562 clean reads, representing 4,045,224 unique sequences. All those sequences were searched against the Rfam and RNAdb databases by BLASTN program [16]. The numbers and proportions of different kinds of small RNA are shown in Table 1. Among the clean reads, 625,232 (6.55%) were found to be miRNA and 6,641,836 (69.6%) were found to be unannotated small RNA, suggesting that barley small RNA hasn’t been mined extensively in previous studies and our study has greater potential to discover more miRNA genes. The rest of the sequences represented non-coding RNA, such as tRNA, rRNA, siRNA, snRNA and other non-coding RNAs.

**Figure 1:**
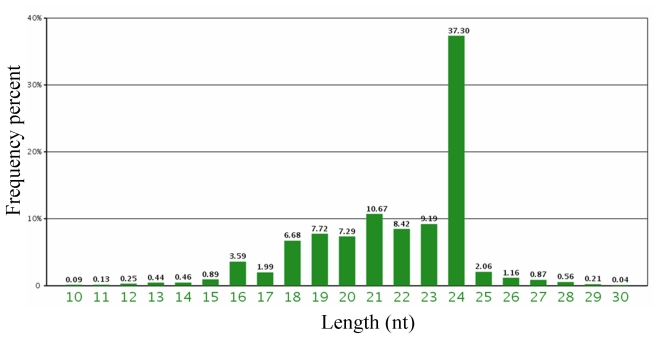
Length, distribution and abundance of barley small RNAs.

Size profile is an important feature to distinguish miRNA from other small RNA and most of the mature miRNAs are of 21–25 nucleotides (nt). The length distribution pattern of the reads was analyzed (as shown in Figure 1). The results indicated that majority of the small RNA from the library were 24 nt in size, accounting for 37.30 % of the total reads (Figure 1), followed by 21 nt (10.67%), 23 nt (9.19%) and 22 nt (8.64%). This distribution pattern is highly consistent with previous other plants small RNA sequencing using Solexa technology, such as *Medicago* [17], rice [18] and peanut [11], as well as *Arabidopsis* where 454 sequencing method was used [19]. It is also consistent with the typical size of Dicer-digestion product. siRNAs of 24 nt in length are known to be involved in heterochromatin modification, especially in a genome with a high content of repetitive sequences [20–21]. The feature of high percentage of 24 nt small RNAs found in barely probably reflects the complexity of the barley genome.

### 2.2. Identification of Conserved Barley miRNA and Evolutionary Conservation

To identify the conserved miRNAs in barley, we analyzed the small RNA sequences with the known plant miRNAs. All high quality small RNA sequences were subject to BLASTN against the known plant miRNAs in miRBase 18.0. When our small RNA dataset was compared with all the available 4742 known plant miRNAs at miRBase database, 126 conserved miRNAs were identified and were categorized into 58 miRNA families allowing one or two mismatches between sequences (Table S1). Solexa sequencing technology is a powerful tool to estimate expression profiles of miRNA genes, which has the ability to generate millions of small RNA sequences, and thus provides a resource to know the abundance of various miRNA families and even distinguish between different members of a given family [10,13]. We analyzed members of the barley miRNA family and found significant difference among them. The miR156 was the largest family with 10 members, followed by miR166 and miR169 each with 9 members, and miR396 with 8 members. Among the remaining 54 miRNAs, 14 families comprised 2–6 members and the rest were of a single member. The frequency of conserved miRNA families in the sequenced library was also analyzed and then used as the index for estimating the relative expression abundance. Among them, 13 miRNA families had more than 1000 sequence counts, implied these miRNAs were highly expressed in barley. The miR167 and miR168 were the most frequently expressed miRNA in our study, with the 165,002 and 94,787 counts, respectively, while miR916, miR5510 and miR5522 were detected only once. Further analysis revealed that there was great variation in read counts of certain members within the same family, suggesting that there was functional divergence within miRNA family. For instance, in miR167 and miR167a family had only 2 counts, while miR167d had 165,002 counts.

To study the evolutionary role of these conserved miRNAs, we performed extensive comparison against known miRNAs in other plant species (Table S1). As expected, most of the miRNAs identified in barley were highly conserved in monocot species, particularly in the Poaceae family. Rice, maize, *Brachypodium* and *Sorghum* shared 28, 16, 15 and 14 orthologs respectively out of the 58 miRNA families in our study. However, it is interesting that barley shared only 5 conserved miRNAs with wheat, suggesting that the identified miRNAs from barley and wheat were not comprehensive enough and extensive mining of more miRNA from them is necessary. Out of the 58 conserved miRNA families, 20 had orthologs in dicot species but not in the monocot counterparts. An implication of these results could be that RNA sampling from different plant tissues and from different developmental stages is necessary for effective coverage and comprehensive miRNA discovery.

### 2.3. Identification of Novel miRNAs

To identify novel miRNAs, we screened all the barley EST and genome sequences for qualified secondary structures (see Material and Methods) following the criteria developed by Meyers *et al*. [22]. In total, 133 small RNAs met the criteria and were considered putative novel barley miRNAs. These miRNAs were classified into 50 families (Table S2). Earlier studies revealed that non-conserved miRNAs are usually expressed at lower levels with tissue or developmental phase-specific pattern [23–24]. In this study, the average novel miRNA member per family was 2.7 and the miRn001 and miRn005 families were found to be quite large, consisting of 15 and 13 members respectively. Furthermore, 27 novel miRNA families possessed a single member while 9 families had 2 members. These results are consistent with the previous studies [15]. At the same time, the expression level of the novel miRNAs was much lower than that of the conserved miRNAs. The precursors of these potential novel miRNAs were identified and formed proper secondary hairpin structures with free energies ranging from −19.2 kcal·mol^−1^ to −154.7 kcal·mol^−1^ (average of −51.60 kcal·mol^−1^) (Table S2). Based upon their secondary structures (Table S3), these miRNAs were considered as the novel barley miRNAs following the plant miRNA annotation criteria [22].

Most importantly, the identification of anti-sense miRNA (miRNA*) from 15 novel miRNA candidates provided more evidence for their existence as novel miRNAs. To investigate the conservation of these 50 novel miRNAs families in a wide range of plant species, all the 148 miRNAs/miRNA* were used as query sequences and BLASTN searched at NCBI. Homologs were found for 70 miRNAs in different plant species, suggesting that the rest of the 78 newly identified miRNAs could be barley-specific.

### 2.4. Validation of Barley miRNA

The expression pattern of the miRNA can provide important information to reveal its function. To examine the potential developmental stage- or tissue-specific roles of miRNAs in barley, we measured the expression level of miRNA in different tissues and different developmental stages using qRT-PCR. Three conserved miRNAs (miR-156d, miR-396d and miR-399a) and three barley-specific miRNAs (miR-n026a*, miR-n029 and miR-n035) were randomly selected to examine their expression pattern using RT-PCR (primers listed in Table S4).

**Figure 2:**
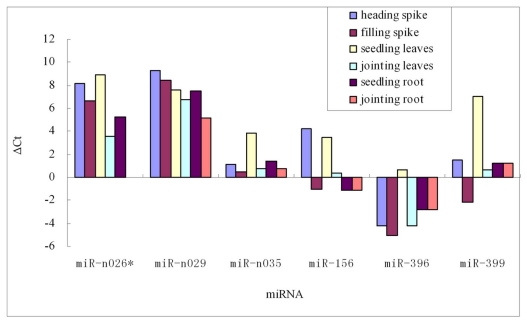
Stem-loop qRT-PCR analysis of barley miRNA in different tissues at different developmental stages.

The results showed that all of the six tested miRNAs were detected in barley (Figure 2), while their expression level varied significantly. The average value of ΔCT of selected three conserved miRNAs was −0.2, which was much lower than that of three novel miRNAs (ΔCT = 4.8), indicating that the conserved miRNA had higher expression compared to the novel miRNA, consistent with our high throughput sequencing results (Table S1). Among novel miRNAs, miR-n026 and miR-n029 had similar expression pattern and both of them showed weak expression in all tissues. miR-n035 was expressed predominantly in filling spike followed by heading spike, root and mature leaves, and weakly in seedling leaves. Among conserved miRNAs, miR-396d appeared to be highly expressed in all tissues, suggesting its primary function in essential biological processes [25]. miR-399b showed lower expression levels in seedling leaves, but was highly expressed in spikes at filling stage, reflecting its possible function as a phosphatase transporter [26]. The miR156 family is a large miRNA family playing important roles in various biological and metabolic processes [27]. According to qRT-PCR results, miRNA156 exhibited higher level of expression in root, young leaves and spikes, but lower in mature leaves and spikes.

### 2.5. miRNA and Stress Tolerance

**Figure 3:**
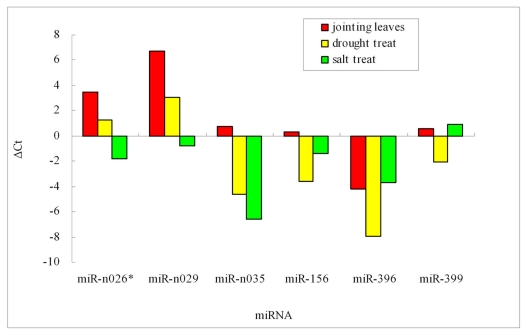
Stem-loop qRT-PCR analysis of barley miRNA in plants exposed to different stresses.

During growth and development, plants are often subject to various abiotic stresses such as salt and drought. Although the roles of many miRNAs in relation to stress response have been found in many plants [28], little is known about the role of barley miRNAs in this respect. In current study, qRT-PCR was conducted to investigate the expression level of randomly selected six miRNAs in normal and stressed (salt or drought) leaves. The results showed that these six miRNAs exhibit different levels of expression with respect to specific stress. miR-n026a*, miR-n029, miR-n035 and miR156d showed higher expression in both drought or salt stressed leaves, respectively, whereas the other two miRNAs, miR396d and miR399b, showed higher expression only in drought stressed leaves. In addition, it was observed that miR-n029 had low expression (perhaps not expressed) in normal plants, while it appeared to be highly expressed in leaves upon drought treatment (Figure 3). This suggested that miRn029 may be a miRNA involved in drought stress-related response in barley. Conserved miRNAs such as miR156d, miR396d and miR399b displayed distinct increase in their expression levels under drought stress, suggesting their involvement in response to drought [29].

### 2.6. Target Gene Prediction

To understand the function of the newly identified barley-specific as well as conserved miRNAs, the putative target sites of these miRNAs were predicted using the psRNATarget program with default parameters (http://plantgrn.noble.org/psRNATarget/). Using the criteria as described in Material and methods, we found 267 potential targets for 44 conserved and 34 novel barley miRNA families, respectively (Table S5). The putative target genes appeared to be involved in a wide range of biological processes and most of them were classified as transcription factors and functional proteins. Similar to other plant species [30,31], most of the conserved miRNAs targeted transcription factors, such as GAMYB transcription factor (hvu-miR159d), nuclear transcription factor (NAC, hvu-miR164a) and CCAAT-binding factor (hvu-miR169d), showing that these conserved miRNAs might play an important role in biological and metabolic processes. Furthermore, some functional proteins, including serine/threonine kinase-like protein and G-box protein, were also found to be the targets of conserved barley miRNAs.

Compared to the conserved miRNA, the targets of novel barley miRNA were mainly enriched in genes involved in protein synthesis. The potential targets included chlorophyll a/b-binding protein, short-chain dehydrogenase/reductases (SDR) and starch branching enzyme (SBE), implying that the novel miRNAs participate in some specific developmental processes in barley. Further analysis of these targets will help for better understanding their function and their regulatory network in barley.

## 3. Experimental Section

### 3.1. Plant Materials

Barley (*H. vulgare* L. variety Clipper) was grown in a growth chamber at a relative humidity of 75% and 26/20 °C day/night temperature with light intensity of 3000 lx. Leaves, stems and roots at four different stages (seedling, jointing, heading and filling), and spikes at heading and filling stages were collected and used for generation of a small RNA library. For expression analysis, seedlings, roots and leaves at jointing stages and spikes were collected. Seedling leaves from plants exposed to 20% PEG and 2% NaCl as drought and salt stresses [32], respectively were also collected and used for expression analysis.

### 3.2. Small RNA Library Preparation and Sequencing

Total RNA was isolated from the roots, stems, leaves and spikes using the TRIzol reagent (Invitrogen, USA) separately, according to the manufacturer's protocol. The RNA quality was examined using gel electrophoresis (28S:18S > 1.5) and Bioanalyzer (Agilent2100, RIN ≥ 8.0). RNA samples from different tissues were pooled together in equal concentration to form single RNA pool. Small RNA with 16–30 nt in length were first separated from the total RNA by size fractionation [33]. After PAGE purification and ligation of a pair of Solexa adaptors to their 5′ and 3′ ends, the small RNA molecules were converted to cDNA by RT-PCR and then the product of RT-PCR was used directly for cluster generation and sequencing analysis using the Illumina Genome Analyzer according to the manufacturer's protocol (BGI, Shenzhen, China). The Illumina FASTQ data generated from this study has been submitted to the NCBI Sequence Read Archive (http://www.ncbi.nlm.nih.gov/sra) under accession number SRX122678.

### 3.3. Bioinformatic Analysis for miRNA Identification

The raw sequences were processed as described by Sunkar *et al*. [34]. After removing the poor-quality reads and adaptor sequences, high quality trimmed sequences (reads with no N, no more than 4 bases whose quality score is lower than 10 and no more than 6 bases whose quality score is lower than 13) with length of 16–30nt were used for further analysis. First, rRNA, tRNA, snRNA, and snoRNA as well as those containing poly-A tail were removed from the small RNA sequences by searching against Rfam database (http://www.sanger.ac.uk/software/Rfam) [35] and NCBI non-coding RNA database (http://www.ncbi.nlm.nih.gov/). Then, all unique sequences were used to do BLASTN search against the miRNA database (miRBase 18.0) [36] to identify conserved miRNAs in barley. Only the perfectly matched sequences were considered to be conserved miRNAs. The criterion previously developed for plant miRNA identification by Meyers *et al.* [22] was used for prediction of potential novel miRNAs. The small RNA sequences which had characteristic fold-back structures could be used to identify precursor sequences for novel miRNAs. The software Mireap (http://sourceforge.net/projects/mireap/) developed by BGI (Beijing Genomics Institute) was used to predict novel miRNA by exploring the secondary structure, the dicer cleavage site and the minimum free energy of the unannotated small RNA tags which could be mapped to barley genome sequence or non-coding EST. Finally, RNA secondary structure was checked using Mfold program [37].

### 3.4. Validation of Barley miRNAs

To validate the predicted miRNAs through the deep sequencing, 3 conserved miRNAs (miR-156d, miR-396d and miR-399b) and 3 barley-specific miRNAs (miR-n026a*, miR-n029 and miR-n035) were randomly selected and subject to qRT-PCR with 18s rRNA as reference. qRT-PCR was also performed to analyze the expression level of these selected six miRNA genes in salt- and drought-stressed barley leaves.

Total RNA isolated from the collected plant materials was used for the first strand cDNA synthesis using one step primeScript miRNA cDNA Synthesis (TaKaRa Bio Inc. Otsu’, Japan) following the manufacturer's protocol. Quantitative real time PCR was performed using the SYBR^®^ Premix Ex Taq™ II kit (TaKaRa, Japan) on Applied Biosystems 7300 Sequence Detection System (Life technology, Foster City, CA, USA). After the reactions were completed, the threshold was manually set (= 0.2) and the threshold cycle (CT) was automatically recorded. (The CT is defined as the fractional cycle number at which the fluorescence signal passes a fixed threshold.) All reactions were performed with three replicates. The ΔCT (CT_sample_ − CT_ref._) was calculated for analyzing miRNAs expression level.

### 3.5. Target Gene Prediction

The potential targets of barley miRNAs were predicted using the psRNATarget program (http://plantgrn.noble.org/psRNATarget/) [38] with default parameters. Newly identified barley miRNA sequences were used as custom miRNA sequences and the barley DFCI Gene index (HvGI) release 11 was used as custom database. All predicted target genes were evaluated by scoring system and the criteria as described by Chi *et al* [39]. Sequences with total score less than 3.0 points were considered to be miRNA targets. Further BLASTX search against the NCBI protein database was further performed to predict functions of potential targets.

## 4. Conclusions

Using high throughput Solexa sequencing of short RNAs, we identified 133 novel and 126 highly conserved miRNAs from a small RNA library constructed by a pool of equal amounts of RNA from multiple tissues, indicating the existence of large number of miRNAs in barley. Randomly selected three novel and three conserved miRNAs were further validated using qRT-PCR, which demonstrates that Solexa sequencing combined with bioinformatic analysis is a powerful tool for miRNA discovery. Among the novel candidate miRNAs, miR-n026* and miR-n028 might play an important role in drought, salt and/or other environmental stress response.

Our comprehensive and systematic approach comparably found higher number of novel as well as conserved barley miRNAs, than previous studies which used only bioinformatics tool and single tissue (leaves) analysis in barley. Our results may provide a solid platform for extensive study of miRNAs’ function in barley and other cereals.

## Figures and Tables

**Table 1:** Distribution of small RNAs among different categories in barley.

Category	Unique sRNA	Percent (%)	Total sRNA	Percent (%)
**Total**	4,045,224	100	9,540,562	100
**miRNA**	23,239	0.57	625,232	6.55
**rRNA**	83,300	2.06	1,006,189	10.55
**repeat**	23,642	0.58	90,970	0.95
**snRNA**	3,020	0.07	15,180	0.16
**snoRNA**	1,872	0.05	6,711	0.07
**tRNA**	16,957	0.42	1,154,444	12.10
**Unannotated**	3,893,194	96.24	6,641,836	69.62
